# Enhanced Surveillance of Coccidioidomycosis, Arizona, USA, 2007–2008

**DOI:** 10.3201/eid1611.100475

**Published:** 2010-11

**Authors:** Clarisse A. Tsang, Shoana M. Anderson, Sara B. Imholte, Laura M. Erhart, Sanny Chen, Benjamin J. Park, Cara Christ, Kenneth K. Komatsu, Tom Chiller, Rebecca H. Sunenshine

**Affiliations:** Author affiliations: Arizona Department of Health Services, Phoenix, Arizona, USA (C.A. Tsang, S.M. Anderson, S.B. Imholte, L.M. Erhart, S. Chen, C. Christ, K.K. Komatsu, R.H. Sunenshine);; Centers for Disease Control and Prevention, Atlanta, Georgia, USA (S. Chen, B.J. Park, T. Chiller, R.H. Sunenshine)

**Keywords:** Coccidioidomycosis, Coccidioides, lung diseases, fungi, central nervous system fungal infections, Arizona, research

## Abstract

Additional public and provider education are needed to reduce delays in diagnosis.

## MEDSCAPE CME

Medscape, LLC is pleased to provide online continuing medical education (CME) for this journal article, allowing clinicians the opportunity to earn CME credit. This activity has been planned and implemented in accordance with the Essential Areas and policies of the Accreditation Council for Continuing Medical Education through the joint sponsorship of Medscape, LLC and Emerging Infectious Diseases. Medscape, LLC is accredited by the ACCME to provide continuing medical education for physicians. Medscape, LLC designates this educational activity for a maximum of 0.5 *AMA PRA Category 1 Credits*™. Physicians should only claim credit commensurate with the extent of their participation in the activity. All other clinicians completing this activity will be issued a certificate of participation. To participate in this journal CME activity: (1) review the learning objectives and author disclosures; (2) study the education content; (3) take the post-test and/or complete the evaluation at www.medscapecme.com/journal/eid; (4) view/print certificate.

## Learning Objectives

Upon completion of this activity, participants will be able to:

- Evaluate the impact of coccidioidomycosis on patients and the broader health system
- Evaluate the performance of a laboratory-based system of diagnosis for coccidioidomycosis and incorporate that information into effective diagnostic strategies.

## Editor

**Carol Snarey, MA,** Technical Writer-Editor, *Emerging Infectious Diseases. Disclosure: Carol Snarey, MA, has disclosed no relevant financial relationships.*

## CME AUTHOR

**Charles P. Vega, MD**, Associate Professor; Residency Director, Department of Family Medicine, University of California, Irvine. *Disclosure: Charles P. Vega, MD, has disclosed no relevant financial relationships.*

## AUTHORS

*Disclosures****: Clarisse A. Tsang, MPH; Shoana M. Anderson, MPH; Sara B. Imholte, MPH; Laura M. Erhart, MPH; Sanny Chen, PhD, MHS; Benjamin J. Park, MD; Cara Christ, MD, MSc; Kenneth K. Komatsu, MPH; Tom Chiller, MD, MPH;*** *and* ***Rebecca H. Sunenshine, MD,*** *have disclosed no relevant financial relationships.*
