# Supplementary material for: Enhanced Surveillance of Coccidioidomycosis, Arizona, USA, 2007–2008
Source: Emerg Infect Dis. 2010 Nov;16(11):1738–44. doi: 10.3201/eid1611.100475 (PMC3294516; doi:10.3201/eid1611.100475)
Supplement: Related texts

# Research article: Enhanced Surveillance of Coccidioidomycosis, Arizona, USA, 2007–2008

**DOI:** 10.3201/eid1611.100475

**Authors:** Clarisse A. Tsang, Shoana M. Anderson, Sara B. Imholte, Laura M. Erhart, Sanny Chen, Benjamin J. Park, Cara Christ, Kenneth K. Komatsu, Tom Chiller, Rebecca H. Sunenshine

**Affiliations:** Author affiliations: Arizona Department of Health Services, Phoenix, Arizona, USA (C.A. Tsang, S.M. Anderson, S.B. Imholte, L.M. Erhart, S. Chen, C. Christ, K.K. Komatsu, R.H. Sunenshine);; Centers for Disease Control and Prevention, Atlanta, Georgia, USA (S. Chen, B.J. Park, T. Chiller, R.H. Sunenshine)

## Abstract

Coccidioidomycosis is endemic to the southwestern United States; 60% of nationally reported cases occur in Arizona. Although the Council of State and Territorial Epidemiologists case definition for coccidioidomycosis requires laboratory and clinical criteria, Arizona uses only laboratory criteria. To validate this case definition and characterize the effects of coccidioidomycosis in Arizona, we interviewed every tenth case-patient with coccidioidomycosis reported during January 2007–February 2008. Of 493 patients interviewed, 44% visited the emergency department, and 41% were hospitalized. Symptoms lasted a median of 120 days. Persons aware of coccidioidomycosis before seeking healthcare were more likely to receive an earlier diagnosis than those unaware of the disease (p = 0.04) and to request testing for *Coccidioides* spp. (p = 0.05). These findings warrant greater public and provider education. Ninety-five percent of patients interviewed met the Council of State and Territorial Epidemiologists clinical case definition, validating the Arizona laboratory-based case definition for surveillance in a coccidiodomycosis-endemic area.

Coccidioidomycosis, or valley fever, is a fungal disease endemic to the southwestern United States, parts of Mexico, and Central and South America. Infection probably occurs when arthroconidia from disrupted soil are inhaled, causing lung infection (*1*,*2*). Signs and symptoms occur 1–4 weeks after exposure and can include fever, cough, fatigue, shortness of breath, headache, joint and muscle aches, and rash. *Coccidioides* spp. can spread to the central nervous system, skin, joints, or bones in <1% of those infected, resulting in extrathoracic dissemination (*3*,*4*).

Each year, an estimated 150,000 persons in the United States become infected with *Coccidioides* spp., and ≈50,000 of these become ill (*5*). Most cases are not reported to health departments, resulting in an underestimated number of coccidioidomycosis cases (*6*). In 2006 in the United States, 8,917 coccidioidomycosis cases were reported, with 5,535 cases reported in Arizona (*7*). The number of reported coccidioidomycosis cases in Arizona began to increase in 1990. From 1990 through 1995, the annual number of reported coccidioidomycosis cases increased from 255 (7/100,000 population) to 623 (15/100,000 population) (*8*). This increase led the Arizona Department of Health Services (ADHS) to change its reporting rules to make coccidioidomycosis a laboratory-reportable illness in 1997.

Since laboratory reporting became mandatory, coccidioidomycosis case reports have rapidly increased in Arizona. In 2006, the number of cases peaked at 5,535 cases (89/100,000 population) and decreased to 4,815 cases (75/100,000 population) in 2007 and to 4,768 cases (73/100,000 population) in 2008. During 2006–2008 in Arizona, the median age of patients with coccidioidomycosis was 52 years (mean 51 years). Fifty-four percent of patients with coccidioidomycosis were male (84/100,000 population), and 46% were female (72/100,000 population).

The Council of State and Territorial Epidemiologists (CSTE) and the Centers for Disease Control and Prevention require laboratory and clinical criteria to meet the case definition for coccidioidomycosis. The laboratory criteria consist of culture, histopathologic, or molecular evidence; or immunologic evidence in the form of detection of immunoglobulin M or immunoglobulin G by immunodiffusion, enzyme immunoassay, latex agglutination, tube precipitin, or complement fixation. Clinical criteria require influenza-like signs and symptoms; pneumonia or other pulmonary lesion; erythema nodosum or erythema multiform rash; involvement of bones, joints, or skin by dissemination; meningitis; or involvement of viscera and lymph nodes (*9*).

Because of Arizona’s large number of cases, ADHS uses only the laboratory component of the CSTE case definition. In 2007, ADHS initiated enhanced coccidioidomycosis surveillance (which included patient interviews) to validate a laboratory-exclusive case definition for coccidioidomycosis and to characterize the effects of the disease on Arizona’s population, healthcare system, and economy. The purpose of this study was to validate this case definition and characterize the effects of coccidioidomycosis in Arizona during January 2007–February 2008.

## Methods

### Study Design

ADHS conducted a population-based investigation of coccidioidomycosis cases reported from January 2007 through February 2008 in Arizona. Every tenth patient with newly identified coccidioidomycosis reported through the statewide surveillance system was sent a letter informing them of the investigation, and all possible methods were used to collect telephone information for each patient. If telephone information was obtained, each selected patient was contacted by telephone and interviewed with the aid of a 15-minute standardized questionnaire. If the patient could not be reached after 3 attempts or refused to be interviewed, the subsequent case-patient was sent an enrollment letter and contacted for an interview. If a patient was <18 years of age, either the parent or guardian was interviewed or the patient was interviewed with a parent or guardian present. Interviewees were asked about the signs and symptoms of coccidioidomycosis they experienced, their healthcare-seeking behavior, their medical treatment information, and the effects of the disease on their daily lives. Interviewees self-reported their race as either white, African American, Asian or Pacific Islander, Native American or Alaska Native, or other and their ethnicity as either Hispanic or non-Hispanic. Interviewees were asked whether they took any immunosuppressive drugs and were provided the following examples: chemotherapy medications, steroids, prednisone, dexamethasone, infliximab, and interferon. If a patient who spoke only Spanish was contacted, a Spanish-speaking interviewer called back to conduct the interview in Spanish.

Symptom information obtained from the interviews was used to determine whether the case-patients met the clinical portion of the CSTE case definition. Case-patients met the CSTE clinical case definition if they had >1 of the following: fever, cough, sore throat, chills, dyspnea, chest pain, hemoptysis, headache, rash, stiff neck, myalgias, or arthralgia.

Questions from the standardized questionnaire were added to the Arizona Behavioral Risk Factor Surveillance System (BRFSS), 2008, an annual population-based telephone survey about health behavior and opinions. Data from BRFSS in 2008 (n = 6,165) were used to represent the general population of Arizona and were compared with data from interviews with coccidioidomycosis patients to understand how the coccidioidomycosis patients’ understanding differed from the BFRSS population’s knowledge of coccidioidomycosis (*10*).

Data from the Arizona Hospital Discharge Database 2007 were used to examine charges for hospitalizations of patients with a primary or secondary diagnosis of coccidioidomycosis (*11*). Hospitalizations were identified by use of the International Classification of Diseases, 9th Revision for coccidioidomycosis (codes beginning with 114).

### Data Management and Analysis

Data were entered into Microsoft Access (Microsoft Corp., Redmond, WA, USA) and analyzed by using SAS software (SAS Institute Inc, Cary, NC, USA). χ^2^ tests were used to detect significant differences between groups, and *t* tests were used to analyze continuous variables. All statistical tests were 2-tailed, and a p value <0.05 was considered significant. Interquartile ranges (IQRs) at the 25th and 75th percentiles were also determined. The positive predictive value of using only the laboratory portion of the CSTE case definition in Arizona was calculated.

## Results

### Study Population

ADHS received reports of 5,664 coccidioidomycosis cases from January 2007 through February 2008. Of the 5,664 reported case-patients, 851 (15%) were sent letters for enrollment, and 493 (9%) were successfully enrolled. Of the 851 patients who received enrollment letters, more than half (493 or 58%) were successfully enrolled; 41 (5%) refused to be interviewed; 15 (2%) were deceased, incapacitated, or incarcerated; 228 (27%) were lost to follow up; and 74 (9%) were in the process of being contacted when the study ended. Interviewed case-patients were similar in age and sex to those case-patients who were not interviewed. However, the number of Native Americans and Hispanics was significantly lower among the interviewed patients than among those not included in the enhanced surveillance population (Table 1).

**Table 1:** Demographic characteristics of reported coccidioidomycosis patients compared with enhanced surveillance patients, Arizona, USA, January 2007–February 2008

Characteristic	Total reported, N = 5,664	Enhanced surveillance, n = 493	p value
Male sex, no. (%)*	3,003 (54)	259 (54)	0.7
Age, y			
Mean	51	52	0.2
Median	52	54	
Range	3 d–100 y	8 mo–100 y	
Race, no. (%)†			
White	1,685 (82)	385 (80)	0.2
African American	158 (8)	33 (7)	0.5
Asian or Pacific Islander	58 (3)	16 (3)	0.6
Native American or Alaska Native	110 (5)	11 (2)	<0.01
Other	37 (2)	37 (8)	<0.01
Unknown	0	1 (0)	
Hispanic ethnicity, no. (%)‡	319 (24)	63 (13)	<0.01

*Sex data were available for only 5,601 reported coccidioidomycosis cases. †In Arizona, race and ethnicity are recorded in separate variables. Therefore, race data do not include a specific category for Hispanic ethnicity but include Hispanics. Race is categorized as White, African American, Asian or Pacific Islander, Native American or Alaska Native, or Other. Race data were available for 2,048 reported coccidioidomycosis patients and for 483 enhanced surveillance patients. ‡In Arizona, race and ethnicity are recorded in separate variables. Ethnicity is categorized as either Hispanic or non-Hispanic. Ethnicity data were available for 1,345 coccidioidomycosis patients and for 482 enhanced surveillance patients.

Comparing data from the interviewed patients with the Arizona population data from the US Census 2000 (*12*), we showed that 449 (91%) of those interviewed had health insurance at the time they sought care compared to 86% of the Arizona population (p<0.01). Interviewed patients differed slightly from BRFSS survey respondents: median age of interviewed patients was significantly lower (54 years vs. 58 years, respectively; p<0.01), and significantly fewer women were interviewed (odds ratio [OR] 1.9, 95% confidence interval [CI] 1.6–2.3). Also, persons with a diagnosis of coccidioidomycosis had lived in Arizona for a median of 12 years compared with BRFSS respondents, who lived in Arizona for a median of 22 years (p<0.01). Among the 493 interviewed patients, 97 (20%) were classified as immunocompromised at the time of illness; 140 (28%) reported a history of heart or lung disease; and 164 (33%) reported having no underlying disease at time of diagnosis (Table 2).

**Table 2:** Clinical and behavioral characteristics of enhanced surveillance coccidioidomycosis patients, Arizona, USA, January 2007–February 2008*

Characteristic	No. (%)
Coexisting condition	
Heart disease	62 (13)
Lung disease	90 (18)
Asthma requiring inhaler	47 (10)
COPD or emphysema	27 (6)
Other	29 (6)
Malignancy	70 (14)
Transplant	11 (2)
HIV	9 (2)
Diabetes mellitus	72 (15)
None	164 (33)
Immunosuppression†	97 (20)
Smoker	
Active	76 (15)
Past	203 (41)
Never	202 (41)
Unknown	12 (2)
Site of infection, n = 282	
Primary pulmonary‡	240 (85)
Disseminated§	42 (15)

*N = 493. COPD, chronic obstructive pulmonary disease. †Immunosuppression is defined as having HIV/AIDS, a solid-organ transplant, or a bone marrow transplant or taking immunosuppressive medications. Immunosuppressive medications refer to medications that suppress the immune system and include chemotherapy medications such as steroids, prednisone, dexamethasone, infliximab, or interferon, as self-reported by patients. ‡Primary pulmonary infection is defined as the lungs being the only site of infection, as self-reported by patients. §Disseminated infection is defined as infection spread to other parts of the body, including the central nervous system, bone, and entire body, as self-reported by patients.

### Effects on Patients

Interviewed coccidioidomycosis patients reported the following common symptoms: fatigue (84%), cough (67%), dyspnea (59%), and fever (54%). Patients sought healthcare a median of 11 days (range 0–2,669 days, IQR 2–31 days) after onset of symptoms. Median time between seeking healthcare and coccidioidomycosis diagnosis was 23 days (range 0–10,280 days, IQR 6–74 days). Thirteen (3%) patients did not know their diagnosis until >2 years after they had seen a doctor for their symptoms.

Patients reported a median of 2 visits (range 0–63 visits, IQR 1–3 visits) to a healthcare provider before coccidioidomycosis testing occurred. Symptoms lasted a median of 42 days (range 0–511 days, IQR 14–65 days) for patients who had recovered at the time of interview (n = 167); 157 days (range 4–5,224 days, IQR 110–277 days) for those who had not recovered at the time of interview (n = 253); and 120 days (range 0–5,224 days, IQR 49–198 days) for the 2 groups combined (n = 420) (Table 3).

**Table 3:** Characteristics of enhanced surveillance coccidioidomycosis patients, and statewide hospital charges identified through the 2007 Arizona Hospital Discharge Database

Description	Mean	Median	Range	Interquartile range	Total
Days from symptom onset to diagnosis date, n = 422	209	55	0–10,280	22–143	
Days from first seeking health care to diagnosis date, n = 422	156	23	0–10,280	6–74	
Days of symptom duration,* n = 420	202	120	0–5,224	49–198	
Days missed from work,† n = 159	31	14	0–365	5–30	
Days missed from school,‡ n = 35	17	9	0–120	3–15	
Days unable to perform daily activities,§ n = 352	96	47	0–1,825	15–120	
Per visit hospital charges for coccidioidomycosis primary diagnoses, n = 1,093	$54,000	$33,000	$113–$1,474,795		$59 million
Per visit hospital charges for coccidioidomycosis primary or secondary diagnoses, n = 1,735	$49,000	$30,000	$113–$1,474,795		$86 million

*Symptom duration analysis includes patients who had recovered and those who had not yet recovered from their symptoms at the time of interview. †Number of days missed from work was available for 159/167 patients who reported that they missed work due to their disease. ‡Number of days missed from school was available for 35/37 patients who reported that they missed school due to their disease. §Number of days patients were unable to perform daily activities was available for 352/369 patients who reported that their disease interfered with their daily activities.

Of the 493 patients interviewed, 225 (46%) were employed and 178 (36%) were retired. Of those employed, 167 (74%) missed a median of 14 workdays (range 0–365 workdays, IQR 5–30 workdays) because of their illness (Table 3). Of interviewed patients, 63 (13%) were attending school when their illness began; 37 (59%) of these students missed a median of 9 days of school due to their illness. When asked about their ability to perform their usual daily activities, 369 (75%) patients said that their illness prevented their performance of usual daily activities at some point during the illness. Among these patients, daily activities were interrupted for a median of 47 days (range 0–1,825 days, IQR 15–120 days) (Table 3).

### Effects on Healthcare and the Economy

Almost half (46%) of the patients interviewed reported >1 visit to the emergency room during the course of illness; 111 (23%) first sought care in an emergency room. Approximately one fourth (26%) of patients visited a healthcare provider >10 times during the course of their illness. Two hundred patients (41%) were hospitalized overnight for their illness, and the median length of hospital stay was 6 days (range 0–306 days, IQR 4–10 days).

Data from the Arizona Hospital Discharge Database (*11*) show that 1,093 hospital visits occurred with a primary diagnosis of coccidioidomycosis in Arizona in 2007, accounting for a total of $59 million in hospital charges and a median of $33,000 per coccidioidomycosis-related hospital visit (Table 3). For the 1,735 visits with a primary or secondary diagnosis of coccidioidomycosis, total charges were $86 million, and the median charge was $30,000 per visit.

### Knowledge of Coccidioidomycosis

Patients who knew about coccidioidomycosis before seeking healthcare were more likely to be diagnosed earlier than those patients who were unfamiliar with the disease (median 20 days [range 0–3,653 days, IQR 6–56 days] vs. 25 days [range 0–10,280 days, IQR 7–144 days], respectively; p = 0.04). Interviewed patients who had prior knowledge of coccidioidomycosis were also twice as likely (95% CI 1.0–3.2, p = 0.05) to request testing for coccidioidomycosis from their healthcare provider. White patients were more likely to have knowledge about coccidioidomycosis before diagnosis than were patients of other racial groups (95% CI 1.7–4.3, p<0.01).

In addition, compared with the general population, interviewed patients were more likely to learn about coccidioidomycosis from their social circles, families, friends, or co-workers (OR 2.3, 95% CI 1.8–2.9). In contrast, those persons contacted through BRFSS were more likely to hear about coccidioidomycosis from the media (TV, radio, newspaper, or Internet) than were persons with coccidioidomycosis (OR 3.3, 95% CI 2.4–4.6). Coccidioidomycosis patients were less likely than BRFSS respondents to have heard about coccidioidomycosis from their doctors (OR 2.2, 95% CI 1.3–3.6).

### Treatment

Antifungal treatment for coccidioidomycosis was prescribed for 303 (61%) patients interviewed. Compared with patients who reported no common symptoms, patients who had symptoms were more likely to be treated with antifungal medication if they reported either chills (68% vs. 57%), shortness of breath (67% vs. 56%), or weight loss (75% vs. 58%; p<0.02 for each). Those with sore throat were significantly less likely to be treated with antifungal medication than those without sore throat (52% vs. 66%, p<0.01). About 60% (289) of patients were treated with antibacterial agents. Of those, 92 (32%) received >1 course of antibacterial drugs (range 2–10 courses, IQR 2–3 courses).

### Comparison to CSTE Case Definition

Of the 493 patients interviewed, 469 (95%) met the CSTE clinical case definition. Thirteen (3%) case-patients reported no symptoms. Ten (2%) had only 1 symptom consistent with coccidioidomycosis but had no symptoms that met the case definition; 1 person had only 1 symptom unrelated to coccidioidomycosis. Case-patients who met the clinical case definition were similar to those who did not meet the case definition in age, race, ethnicity, and gender.

## Discussion

This investigation provides the largest population-based estimate of the effects of coccidioidomycosis in Arizona. We identified substantial personal and economic costs due to coccidioidomycosis among Arizonans with respect to duration and severity of illness, healthcare use, and healthcare costs. We also found marked delays in diagnosis as well as long duration of symptoms.

In our cohort, persons with coccidioidomycosis had prolonged symptoms for a median of 120 days, substantially longer than previous reports that indicated that coccidioidomycosis symptoms typically last <21 days (fatigue may last longer) (*2*,*13*). A study conducted among US Navy SEALS, a presumably healthy and relatively young population, reported median symptom duration of 19 days (range 2–63 days) (*14*). Our investigation identified a high number of missed workdays (median 14 days, range 0–365 days, IQR 5–30 days) and days during which persons could not perform their daily activities (median 47 days, range 0–1,825 days, IQR 15–120 days). In a study among military members, persons with coccidioidomycosis lost an average of 35 days from work (*15*). These data support the finding that coccidioidomycosis greatly affects a person’s ability to function and remain productive once the disease develops.

In addition, we found a substantial delay between symptom onset and disease diagnosis. The delay in seeking medical care needs to be addressed by increasing public education about the signs and symptoms of coccidioidomycosis and the importance of seeking care early to obtain an accurate diagnosis. Our data show that the delay between seeking healthcare and ordering a diagnostic test may also be shortened by patient education. Persons who knew about coccidioidomycosis before seeking healthcare were more likely to request coccidioidomycosis testing and were more likely to receive a diagnosis earlier than those who were not familiar with the disease. Our data show that 46% of patients sought medical care without a fever, making recognition of the disease difficult for physicians and patients and possibly contributing to delays in diagnosis. Additionally, the nonspecific manifestation of respiratory illness in coccidioiodomycosis patients is indistinguishable from the manifestation of community-acquired pneumonia, which makes accurate diagnosis even more difficult (*16*).

Besides the effects on patients, this disease greatly affects the healthcare system. In our investigation, ≈25% of patients visited a healthcare provider >10 times during the course of their illness, and 41% of all interviewed patients were hospitalized. In 1993, a study conducted by Kerrick et al. showed that college students who had coccidioidomycosis visited their doctor an average of 7 times before the disease resolved (*17*). Similarly, Leake et al. found that patients >60 years of age had a median of 4 medical visits (range 1 to >30 visits) during the course of their illness (*18*). In this same study, 59% of patients were hospitalized for a median of 7 days. A study among military personnel by Crum et al. found that 22% of those with pulmonary disease and 40% with disseminated disease were hospitalized (*15*). Our study is consistent with this literature, but, being population-based, is more representative of the disease’s effects on Arizona residents.

In addition to the costs generated by excess healthcare visits, we found the costs associated with hospitalizations to be higher than costs found in previous studies. Our data show hospital charges totaling $86 million (mean $49,000 per hospitalization) among Arizona patients who had primary or secondary diagnoses in 2007. This total is much higher than that found in a previous analysis, which showed total annual hospital charges of $2 million in 1998, increasing to $19 million in 2001 (*19*). Our data clearly show the growing costs of coccidioidomycosis and its effect on healthcare costs in Arizona.

Persons with a diagnosis of coccidioidomycosis reported living in Arizona for significantly fewer years (median 12) than a sample of the general population (median 22). This finding is consistent with previous studies, which showed that relatively recent relocation to Arizona from a non-disease–endemic area is a risk factor for developing the disease (*18*,*20*,*21*). Leake et al. examined patients >60 years of age and identified a median duration of residence in Arizona of 6.5 years for coccidioidomycosis patients compared with 19.5 years for controls from the same geographic area (*18*).

We also found that the modified surveillance case definition used by Arizona is appropriate and has a high positive predictive value for the population in this coccidioidomycosis-endemic area. Arizona originally adopted a modified coccidioidomycosis case definition that includes only the laboratory criteria for several reasons. First, clinical information is rarely reported to public health agencies, and with >4,000 cases reported each year, obtaining this information for each case is resource intensive. Second, our experience suggests that most *Coccidioides* tests are performed on symptomatic patients (i.e., persons sick enough to seek medical attention). The data from this investigation confirm that our modified case definition is highly specific: 95% of cases reported to ADHS met both the laboratory and clinical criteria specified in the CSTE definition; the other 5% either had no symptoms or had symptoms that were inconsistent with the coccidioidomycosis case definition. These findings suggest that eliminating clinical criteria from the coccidioidomycosis case definition allows for simpler surveillance methods and requires fewer resources yet still accurately estimates prevalence and incidence of the disease in endemic regions.

Our investigation has several limitations. Coccidioidomycosis surveillance in Arizona requires a laboratory diagnosis of the disease. Because patients without a laboratory-confirmed diagnosis are missed, the number of reported coccidioidomycosis cases underestimates the actual number of cases. These reports come from outside sources, so a minor chance of error in data collection exists. Additionally, because case-patients reported to the health department are usually persons who are sick enough to seek medical attention for their symptoms and receive testing, this study is biased toward more patients with severe coccidioidomycosis cases and toward those with medical insurance. Furthermore, this enhanced surveillance data relied on self-reporting by patients. Because patients often lack medical knowledge or may refuse to answer questions during the interview, information reported may be missing or inaccurate. For instance, we relied on self-reported use of immunosuppressive medications, and these data were not verified by medical records or by physicians.

Self-reporting is subject to recall bias. However, most case-patients were contacted within a few months of their diagnosis, minimizing recall bias as much as possible. In addition, because telephone interviews were conducted, the data are limited to information from persons who were at home and who had telephones. These factors might explain the underrepresentation of Native American and Hispanics in the enhanced surveillance cohort, although every effort was made to capture information from persons who spoke only Spanish. Table 1 shows that Native Americans were underrepresented in the enhanced surveillance sample compared with the statewide coccidioidomycosis cases reported from January 2007 through February 2008 (2% vs. 5%; p<0.01), a difference possibly caused by lack of telephone contact information in this community. Another possible contributor to the underreporting of Native Americans is the fact that tribal entities are not required to report coccidioidomycosis cases to the state health department; however, most tribes in Arizona do voluntarily report infectious diseases to ADHS. Last, the data from the BRFSS survey may not accurately reflect the general population because respondents tended to be older and were more likely to be female than the Arizona population reported in the US Census 2000 (*22*).

This population-based cohort investigation illustrates the severe effects that coccidioidomycosis has on patients, the healthcare system, and the economy in Arizona. The data emphasize the need for effective education campaigns aimed at the general public and healthcare providers to decrease delays in diagnosis of coccidioidomycosis, which would probably reduce unnecessary use of antimicrobial drugs, relieve patient anxiety, and enable early recognition and treatment of the disease. Furthermore, the data validate a case definition that uses only laboratory criteria for coccidioidomycosis surveillance in disease-endemic areas. This information could be used to propose changes to the national CSTE coccidioidomycosis case definition in other US disease-endemic areas and thus reduce resources needed for an accurate assessment of the extent of the disease and its effects.

## References

[1] Stevens DA. Coccidioidomycosis. N Engl J Med. 1995;332:1077–82. 10.1056/NEJM1995042033216077898527

[2] Chiller TM, Galgiani JN, Stevens DA. Coccidioidomycosis. Infect Dis Clin North Am. 2003;17:41–57. 10.1016/S0891-5520(02)00040-512751260

[3] Kirkland TN, Fierer J. Coccidioidomycosis: a reemerging infectious disease. Emerg Infect Dis. 1996;2:192–9. 10.3201/eid0203.9603058903229PMC2626789

[4] Ampel NM. The complex immunology of human coccidioidomycosis. Ann N Y Acad Sci. 2007;1111:245–58. 10.1196/annals.1406.03217363432

[5] Galgiani JN, Ampel NM, Blair JE, Catanzaro A, Johnson RH, Stevens DA, Coccidioidomycosis. Clin Infect Dis. 2005;41:1217–23. 10.1086/49699116206093

[6] Doyle TJ, Glynn M, Groseclose S. Completeness of notifiable infectious disease reporting in the United States: an analytical literature review. Am J Epidemiol. 2002;155:866–74. 10.1093/aje/155.9.86611978592

[7] Centers for Disease Control and Prevention. Summary of notifiable diseases—United States, 2006. MMWR Morb Mortal Wkly Rep. 2008;55:1–94.18354375

[8] Centers for Disease Control and Prevention. Coccidioidomycosis—Arizona, 1990–1995. MMWR Morb Mortal Wkly Rep. 1996;45:1069–73.8975119

[9] Centers for Disease Control and Prevention. Case definitions for infectious conditions under public health surveillance, coccidioidomycosis [cited 2008 Oct 3]. http://www.cdc.gov/ncphi/disss/nndss/casedef/coccidioid2008.htm

[10] Centers for Disease Control and Prevention. CDC’s behavior risk factor surveillance system. 2008 [cited 2010 Mar 30]. http://www.cdc.gov/BRFSS/

[11] Bureau of Public Health Statistics, Arizona Department of Health Services. Arizona hospital discharge data set(s), 2003–2007 [cited 2010 Mar 30]. http://www.azdhs.gov/plan/crr/ddr/index.htm

[12] Mills RJ. Health insurance coverage: 2000. US Census Bureau. 2001 Sep [cited 2009 Jul 27]. http://www.census.gov/prod/2001pubs/p60-215.pdf

[13] Saubolle MA, McKellar PP, Sussland D. Epidemiologic, clinical, and diagnostic aspects of coccidioidomycosis. J Clin Microbiol. 2007;45:26–30. 10.1128/JCM.02230-0617108067PMC1828958

[14] Crum N, Lamb C, Utz G, Amundson D, Wallace M. Coccidioidomycosis outbreak among Untied States Navy SEALs training in a *Coccidioides immitis*–endemic area—Coalinga, California. J Infect Dis. 2002;186:865–8. 10.1086/34240912198626

[15] Crum NF, Lederman ER, Stafford CM, Parrish JS, Wallace MR. Coccidioidomycosis: a descriptive survey of a reemerging disease. Clinical characteristics and current controversies. Medicine. 2004;83:149–75. 10.1097/01.md.0000126762.91040.fd15118543

[16] Valdivia L, Nix D, Wright M, Lindberg E, Fagan T, Lieberman D, Coccidioidomycosis as a common cause of community-acquired pneumonia. Emerg Infect Dis. 2006;12:958–62. 10.3201/eid1206.06002816707052PMC3373055

[17] Kerrick SS, Lundergan LL, Galgiani JN. Coccidioidomycosis at a university health service. Am Rev Respir Dis. 1985;131:100–2.396669510.1164/arrd.1985.131.1.100

[18] Leake JA, Mosley DG, England B, Graham JV, Plikaytis BD, Ampel NM, Risk factors for acute symptomatic coccidioidomycosis among elderly persons in Arizona, 1996–1997. J Infect Dis. 2000;181:1435–40. 10.1086/31540010753733

[19] Park BJ, Sigel K, Vaz V, Komatsu K, McRill C, Phelan M, An epidemic of coccidioidomycosis in Arizona associated with climatic changes 1998–2001. J Infect Dis. 2005;191:1981–7. 10.1086/43009215871133

[20] Smith CE, Bear RR, Rosenberger HG, Whiting EG. Effect of season and dust control on coccidioidomycosis. J Am Med Assoc. 1946;132:833–8. 10.1001/jama.1946.0287049001100320274881

[21] Stern NG, Galgiani JN. Coccidioidomycosis among scholarship athletes and other college students, Arizona, USA. Emerg Infect Dis. 2010;16:321–3.2011357110.3201/eid1602.090918PMC2958011

[22] U.S. Bureau of Census. Profile of general demographic characteristics: 2000. 2000 [cited 2010 Mar 11]. http://censtats.census.gov/data/AZ/04004.pdf

# Research article: Enhanced Surveillance of Coccidioidomycosis, Arizona, USA, 2007–2008

**DOI:** 10.3201/eid1611.100475

**Authors:** Clarisse A. Tsang, Shoana M. Anderson, Sara B. Imholte, Laura M. Erhart, Sanny Chen, Benjamin J. Park, Cara Christ, Kenneth K. Komatsu, Tom Chiller, Rebecca H. Sunenshine

**Affiliations:** Author affiliations: Arizona Department of Health Services, Phoenix, Arizona, USA (C.A. Tsang, S.M. Anderson, S.B. Imholte, L.M. Erhart, S. Chen, C. Christ, K.K. Komatsu, R.H. Sunenshine);; Centers for Disease Control and Prevention, Atlanta, Georgia, USA (S. Chen, B.J. Park, T. Chiller, R.H. Sunenshine)

## Earning CME Credit

To obtain credit, you should first read the journal article. After reading the article, you should be able to answer the following, related, multiple-choice questions. To complete the questions and earn continuing medical education (CME) credit, please go to www.medscapecme.com/journal/eid. Credit cannot be obtained for tests completed on paper, although you may use the worksheet below to keep a record of your answers. You must be a registered user on Medscape.com. If you are not registered on Medscape.com, please click on the New Users: Free Registration link on the left hand side of the website to register. Only one answer is correct for each question. Once you successfully answer all post-test questions you will be able to view and/or print your certificate. For questions regarding the content of this activity, contact the accredited provider, CME@medscape.net. For technical assistance, contact CME@webmd.net. American Medical Association’s Physician’s Recognition Award (AMA PRA) credits are accepted in the US as evidence of participation in CME activities. For further information on this award, please refer to http://www.ama-assn.org/ama/pub/category/2922.html. The AMA has determined that physicians not licensed in the US who participate in this CME activity are eligible for *AMA PRA Category 1 Credits*™. Through agreements that the AMA has made with agencies in some countries, AMA PRA credit is acceptable as evidence of participation in CME activities. If you are not licensed in the US and want to obtain an AMA PRA CME credit, please complete the questions online, print the certificate and present it to your national medical association.

## Article Title: Enhanced Surveillance of Coccidioidomycosis, Arizona, USA, 2007–2008

## CME Questions

**1. You are seeing a 52-year-old man in Arizona who has a history of cough and intermittent fever for 2 months. He was previously seen in an urgent care center and an emergency department, and he completed one course of azithromycin with little effect.You suspect that this man might have coccidioidomycosis. Which of the following statements regarding the definition of coccidioidomycosis in the current case series is most accurate?**A. Only clinical criteria were important in the definition of coccidioidomycosisB. The vast majority of laboratory-diagnosed cases met the clinical criteria for coccidioidomycosisC. Both clinical and laboratory criteria were required to define coccidioidomycosisD. Fungal culture was the sole means of laboratory diagnosis of coccidioidomycosis**2. Which additional symptom in this gentleman would support a clinical suspicion of coccidioidomycosis according to the current study?**A. FatigueB. CoughC. DyspneaD. F ever**3. The patient from question 1 is diagnosed with coccidioidomycosis, and he is worried what this diagnosis might cost him financially and in terms of his daily function. Based on the results of the current study, what can you tell him?**A. Most patients with coccidioidomycosis do not miss work because of illnessB. Three quarters of patients report an impact on usual daily activitiesC. Less than 10% of patients visit the emergency departmentD. Hospitalization for coccidioidomycosis is exceedingly rare**4. Which of the following factors from the current study had the biggest impact on being diagnosed and treated for coccidioidomycosis earlier?**A. Education about coccidioidomycosis from television announcementsB. Education about coccidioidomycosis from newspaper campaignsC. Education about coccidioidomycosis from social circles or familiesD. The presence of sore throat

## Activity Evaluation

**Table:** 

1. The activity supported the learning objectives.				
Strongly Disagree				Strongly Agree
1	2	3	4	5
2. The material was organized clearly for learning to occur.				
Strongly Disagree				Strongly Agree
1	2	3	4	5
3. The content learned from this activity will impact my practice.				
Strongly Disagree				Strongly Agree
1	2	3	4	5
4. The activity was presented objectively and free of commercial bias.				
Strongly Disagree				Strongly Agree
1	2	3	4	5
